# Editorialets

**Published:** 1909-11

**Authors:** 


### Editorialets.

---

TWO DOLLARS FOR ONE.—Doctor, it is a luxury to shave yourself with a good safety razor. You can do it in three minutes; can't cut yourself. I wouldn't take $100 for mine if I couldn't get another. I will send you one post paid on receipt of order enclosing $1 for a year's subscription to the Famous "Red Back." Renewals count if arrears are paid. Silver plated, guaranteed five years. Four Keen Cutter blades go with it. "Do it now."

---

THE NEW MEMBER of the State Board of Health is Dr. J. T. O'Farrell, of Richmond, Texas, appointed in place of Dr. E. B. Parsons, of Palestine, whose appointment we announced in our last issue. Dr. Parsons felt bound to resign, as he holds an important salaried position as railroad surgeon of the I. & G. N.

---

DR. J. H. FLORENCE having resigned from the quarantine service to accept the office of Medical Director of the great Southern Life Insurance Company, has removed to Houston. Dr. R. W. Knox, of Houston, has accepted the appointment of Assistant Medical Director of the company. The company is to be sincerely congratulated upon their wisdom in selecting, as well as their luck in securing, these exceptionally high class officers.

---

WILL some interested publisher kindly send me the address of The Medical Equipment Company, a Mr. Henderson, manager, lately at 127 East 123rd Street, New York.

---

THE TEXAS ASSOCIATION OF HEALTH OFFICERS held a large and enthusiastic meeting in Austin October 7 and 8 (ult.), at which were present more than one hundred health officers, representing every section of the State. Many interesting papers were read, most of which will be published by the State Public Health Department. The occasion of the meeting was to draft and agree upon what is termed the Advisory Code of Sanitary Rules, as distinguished from the Mandatory Code provided for by law, and which has been perfected by the State Board of Health and will soon be promulgated. The Advisory Code will not have the force of law until or unless it is approved by the next Legislature, but it is expected that the municipal governments of the larger cities will enact it into ordinances and enforce it as far as practicable. The men engaged in this altruistic work who assembled here on that occasion were earnest, zealous, even enthusiastic in the cause, and the Association should and no doubt will have the cordial and earnest co-operation and backing of all good citizens. There never was a more unselfish movement inaugurated.

---

DR. LYDSTON'S paper published in our October issue created a sensation, as usual. Orders for it have long since exhausted the edition, and are still being received. One firm in Iowa, not advertisers in the "Red Back," ordered one hundred copies. Hence I feel obliged to reproduce it.

Apropos of Dr. Lydston, in pursuance of his campaign against the medical despotism of the Octopus that has grown up in the United States and in defense of professional liberty, fairness and decency, and clean medical politics, he has begun the publication of a series of articles calling attention to some of the many evils of the A. M. A., as at present conducted. He appeals to the independent medical press to aid him in the battle by the publication and quotation of his articles simultaneously in a number of journals.

---

In another part of this issue will be found an article from a Cleveland, Ohio, paper, the re-opening of the campaign, as it were, a sort of truce having followed the gorgeous calcimining of The Dictator at Atlantic City. Next month I will reproduce my "Chronicles of the Octopus" and "Song of the First Lord," by request. They all want it.

---

Public Health Reports.—Valuable documents for free distribution. The M. H. S. and Public Health Bureau has such a number of valuable reports which can be had for the asking. We have received the following:

"The Surface Privy as a Factor in Soil Pollution, with Resulting Hook-Worm Disease and Typhoid Fever," Styles.

"The Fixing Power of Alkaloids on Volatile Acids, Etc.," Elvooe.

"The Influence of Certain Drugs Upon the Toxicity of Acetanilide and Antipyrene," Hale.

"Hook-Worm Disease in Relation to the Negro," Styles.

"Report on the Twelfth International Congress on Alcoholism, and the Sixteenth International Medical Congress," Reid Hunt.

Dr. Hunt, Chief of Division of Pharmacology, Hygienic Laboratory, U. S. P. H. & M. H. S., was detailed to attend these Congresses, and the above is his report. It is intensely interesting. It is very gratifying to the editor of the Texas Medical Journal to see that Dr. Hunt says that "The consensus of opinion of the speakers seemed to be that alcohol, in any form, is but seldom of distinct value in the treatment of disease, and some evidence was brought forward to show that alcohol even in moderate amounts has an unfavorable effect upon offspring and has a tendency to lower resistance to infection. The dangers of alcohol to those with any tendency to nervous or mental diseases was especially emphasized by Dr. F. W. Mott, and the effects upon children by Professor Clouston."

---

Dr. Otto J. Stein, who is President of the American Academy of Oto-Laryngology, has been elected director on the board of the Post-Graduate Medical School of Chicago. Also Dr. Emil

Ries has been elected secretary to fill the vacancy caused by resignation of Dr. Franklin H. Martin.

---

DR. FRANZ KOEMPEL, a prominent New York surgeon, operating at the Woman's Hospital, makes the most interesting statement that during the last six months the new antiseptic, Chinosol, has been his sheet anchor, and that he has entirely discarded the use of carbolic acid and bichloride of mercury.

---

THE Great Lombroso is dead. Died at Turin, October 18, aged 73.

---

THE GOVERNOR declines to carry into effect the recent act of the Legislature appropriating $40,000 for a Leprosarium in Texas and a $3000 salary for a superintendent. I don't know but what he is right about it. For six or eight lepers, that looks like "going some," especially as the disease is not contagious nor infectious. Tuberculosis is infectious and the infection is being scattered everywhere, but His Excellency vetoed the bill for a corral for them as a protection of the public against the deadly infection.

---

Dr. G. H. Wooten, Dr. F. A. Maxwell and Dr. Joe S. Wooten have recently become associated with Dr. T. O. Maxwell in the Texas School and Sanitarium for Defectives at Austin, and will in future assist in conducting the affairs of the institution.

